# The Planning of Difficulty Curves in an Exergame for Inhibitory Control Stimulation in a School Intervention Program: A Pilot Study

**DOI:** 10.3389/fpsyg.2019.02271

**Published:** 2019-10-15

**Authors:** João B. Mossmann, Bernardo B. Cerqueira, Débora N. F. Barbosa, Rochele P. Fonseca, Eliseo B. Reategui

**Affiliations:** ^1^Instituto de Ciências Criativas e Tecnológicas, Feevale University, Novo Hamburgo, Brazil; ^2^Grupo Neuropsicologia Clínica e Experimental, PUCRS, Porto Alegre, Brazil; ^3^Programa de Pós-Graduação em Informática na Educação, UFRGS, Porto Alegre, Brazil

**Keywords:** digital games, difficulty curve, Inhibitory Control, exergames, Apollo & Rosetta

## Abstract

Apollo & Rosetta is an Exergame developed for Inhibitory Control stimulation in Elementary School children. This works’ goal has been to demonstrate the behavior of the difficulty curves planned for seven activities (minigames) ingame, as well as their correspondence with the variables collected during a pilot neuropsychological intervention. Seven students participated in the study and played the minigames 1528 times during the 3-month intervention. Each of the minigames had a difficulty curve computed with the goal of keeping the players in the state of Flow. The curves were designed in cycles which grow throughout levels (Normal Level) to a peak (Peak Level), followed by a rest period (Rest Level). The pilot study encompassed three different analyses: (1) Exploratory performance analysis with Spearman correlation, which indicated a positive and significant general correlation between performance and level difficulty; (2) Success exploratory analysis, which showed that as the stages progressed, the success rate increased, even if the level difficulty also increased; (3) Analysis of the factors which influenced performance, through Mixed Effects Logistic Regression and the Backward method. This analysis demonstrated that the odds ratio for overcoming challenges between Normal levels was 0.71 [0.59;0.86] times lower than Rest Level (*p*-value = 0.000), whereas in Peak levels it was 0.62 [0.47;0.83] times lower than Rest level values (*p*-value = 0.001). These data confirm the overall planned behavior of the difficulty curves.

## Introduction

Executive Functions are the most complex cognitive abilities that manage control-demanding tasks and are essential for thoughts and behavior regulation in order to achieve goals (Friedman and Miyake, 2017). Inhibitory Control (IC), one of the components of Executive Functions, is the ability to perform behavior control, and also to stop inappropriate actions/behaviors. It allows a person to choose how to react and behave in a given situation (Miyake et al., 2000; Carlson and Wang, 2007; Diamond, 2013, 2015). Self-control (Zelazo, 2015) and emotional understanding of oneself and of others are also associated with IC (Rueda and Paz-Alonzo, 2013). IC is also known to be related to students’ academic performance (Brock et al., 2009; Visu-Petra et al., 2011). Furthermore, children with Attention Deficit Hyperactivity Disorder (ADHD) have impairments concerning IC (Salum et al., 2014). Currently, difficulties have been found in the reproduction of research results related to the use of computer programs, such as digital games, for IC stimulation. This demonstrates the need for further evidence-based investigation (Diamond and Lee, 2011; Diamond and Ling, 2016).

This article presents a study about the computation of difficulty curves (DC) for an exergame designed for IC stimulation. Exergames are computer programs in which the body is the element of interaction between the player and the game (Staiano and Calvert, 2011). Our goal here has been to design DCs that would keep the players in the state of Flow (Csikszentmihalyi, 2014), a condition achieved when people are fully focused on their activities.

The exergame, called *The Incredible Adventures of Apollo & Rosetta in Space* (A&R) (Mossmann et al., 2017), is tailored to Elementary School children. Seven different activities (minigames) in the game allow the player to deal with different IC stimulation events. Each activity presents a specific DC designed to generate a gradual increase in executive difficulties. It also presents challenges in order to balance cognitive stimulation, fun, engagement, and physical fatigue.

This article explains the design and implementation of a model for the computation of the exergame’s DCs. It also presents the results of a pilot neuropsychological intervention that took place in a school environment.

## Apollo & Rosetta (A&R)

Apollo & Rosetta was designed as an exergame for the IC stimulation, conceived and developed by a multidisciplinary team and evaluated by specialists from the EF field. The ludic narrative developed in the game has a space fiction theme designed for Elementary schoolchildren. Each of the seven activities in the game, structured as minigames, was created to perform different types of IC stimulation, as detailed in Mossmann et al. (2017). The activities were divided into three groups:

*Seriated activities:* (1) **Jumping Asteroids** is a game in which the player sees four asteroids and must jump over a colored pair, which changes color in each round. If the color matches those in a list, the player must not step on the colored pair anymore. (2) **Deciphering codes** is a game in which the player must place his/her hands or feet on the specified places. However, a character may occasionally say a word, which is a determinant of whether the player should keep doing the same or perform another movement.
*Activities with distractors:* (3) **Explorer**, a game in which the player must move laterally to guide the character in a path, and collect what is indicated in a list while collectible items and distractors, that must be dodged, arise; (4) **Stellar Laboratory** is a game in which, using one’s feet and hands, one must collect colored and numbered items that match the corresponding colored and numbered buttons on the screen; (5) **Challenge of the Opposites** is a game in which the player must collect items using his/her hands or feet. The player is guided by sound instructions and, at any given moment, he/she must do the opposite of what is instructed.
*Prepotent motor response inhibition activities:* (6) **Particle Accelerator Tunnel**, in which the player must move laterally to dodge obstacles and, at any given moment, move in a direction that is contrary to the usual; and (7) **Galactic Art**, in which the player must hit colored flying balls with his/her hand, and refrain from action when they are white/black. In addition, the player must attempt to scare away space flies that occasionally invade the screen.

### Development and Quantification of the Difficulty Curves

Apollo & Rosetta was developed according to the Flow model (Csikszentmihalyi, 2014) to increase children’s fun and consequently their engagement in the game. The Flow state is reached when people are fully focused on their current activity, enabling them to achieve their top performance level. For this state to be achieved there needs to be a balance between the challenge and the person’s ability to carry out the given activity. In the context of games, this theory has been used by game designers in an attempt to create engaging games (Cowley et al., 2008).

Among the existing techniques to develop a game with a balanced DC, Schell (2008) states that the difficulty must be increased progressively each time the player performs a successful action. A&R employed a variation of a methodology commonly used in the digital game industry (Schell, 2008; McMillan, 2013), which consists of assigning numerical variables related to the difficulty level and the quantification of the execution of the existing game mechanics^1^ (GM).

During the development of the A&R game, evaluation steps were carried out to evaluate its gameplay and usability (Mossmann et al., 2017), in which the priority was to collect information based on the assumptions of the Flow model. The data collected in the sessions indicated aspects that could control as well as contribute to a balanced experience between the challenges presented and the individual’s ability (Cowley et al., 2008). Cycles of nine levels were designed for the functioning of the curve, as detailed in section “Model Application.”

The operation of the DC has been based on a numerical scale varying from 0 to 10. On this scale, each GM received a value related to its difficulty, respecting the fact that the sum of the values assigned to all the GM had to be 10. Thus, the recurrence of each GM and the effort required to overcome each challenge is what varies among the levels, so that the game designer may compute, manipulate, and extend the DC as much as desirable. Therefore, a different weight was attributed to each GM related to the activity. Then, to design the difficulty of a level, a value was assigned to each GM, according to the following equation:

d=w*y

TD=d1+d2+…+dn

while d is the difficulty of a given GM, w is the representative weight of that difficulty, and y is the intensity in which the GM will be present at the game level. In this context, the level of a game is composed of a sequence of GM. Therefore, the difficulty must be the sum of all the GM (d) that make up a certain level, while the Total Difficulty (TD) is the result of this sum.

#### Model Application

To explain the model of difficulty quantification, the minigame *Particle Accelerator Tunnel* is used as an example. The purpose of this activity is to guide (1) the character through a tunnel, as the player moves his/her body to the right or to the left. Throughout the tunnel, there are obstacles that the player must dodge (2). Thus, the player must guide the character, preventing his collision with the obstacles on the way. There are two view modes ingame: In the first one, the game character appears on his back, so the player’s laterality coincides with that of the character’s. In the second one, the camera rotates, giving the player a frontal view (3) of the character for a few seconds. Therefore, the player must guide (4) the character having as reference his/her laterality (the player’s left side is the character’s right side, and the player’s right side is the character’s left side), inhibiting the tendency to move in the usual way to avoid obstacles (Mossmann et al., 2017). These GM were separated as follows:

• Speed (1): Character’s speed.
• Obstacle Quantity (2): the number of dodgeable obstacles generated in the level.
• Inverted Camera Distance (3): the distance between the camera and the character, which increases according to his/her speed.
• Reverse obstacle quantity (4): the number of obstacles generated during the camera inversion.

**FIGURE 1:**
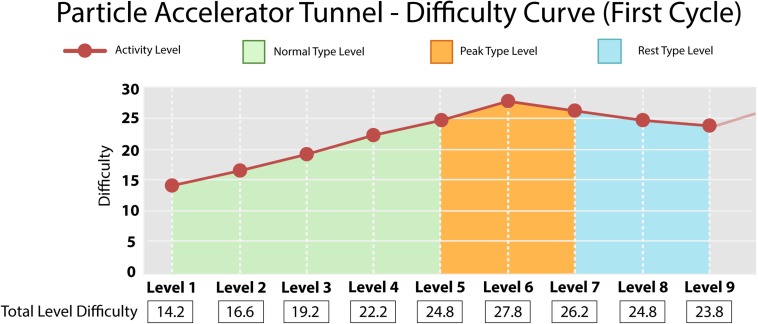
Graph representing the evolution of difficulty of the first cycle (nine levels) of the Particle Accelerator Tunnel minigame. Activity levels and DCs are represented in a reddish color. The graph starts at “14.2” due to the sum of the player’s required GM at the given level, considering level 1 as the minimum and initial value of a DC. Normal level types, on which the difficulty has a gradual increase, are pictured in the green range for five levels. The peak level type is represented in the orange range at the sixth level and always follows the growth interval and precedes the rest interval. The resting level types are characterized by the gradual decrease of the difficulty of the three levels – represented in the blue interval – after the maximum difficulty of that cycle and precede the next cycle, which maintains the growth pattern of the DC detailed here.

Figure 1 shows how the values were distributed in each level type: Normal (1, 2, 3, 4, and 5), Peak (6), and Rest (7, 8, and 9). The last type has a TD value that is lower than those of the Peak type to provide a moment of rest for the player, keeping him as close as possible to the Flow state. Thus, it was crucial to plan the difficulty values according to this tension relief context. Figure 1 also presents the first cycle of the DC of this minigame. The TD column is computed by multiplying the value of the GM by its weight and adding each result, as in the Level 1 (1.1 × 4) + (5 × 1) + (0.9 × 2) + (1 × 3) = 14.2 (Supplementary Table 1), followed by the evolution of the difficulty of the first cycle, demonstrating the peak and the rest levels.

To define the changes in the subsequent cycles, the values from the next cycle (level 10 – the first level of the second cycle) are increased, so that it has a TD greater than or equal to level 6 (the peak of the previous cycle). These relationships were created to standardize the curve’s behavior.

## Materials and Methods

The design of the A&R school intervention as a pilot study followed a cross-sectional approach (Shaughnessy et al., 2012). The pilot study was carried out in a private school located in Novo Hamburgo (Rio Grande do Sul, Brazil), both school and participants were selected by convenience sampling. The game activities were conducted out of the class hours so as not to interfere with the students’ curricular activities. The exergame was used by elementary school children. A total of seven participants joined the study and played a total of 1528 rounds (*n* = 1528) of the game. The school intervention program was carried out in 25 sessions of 20 min, three times a week for 3 months. While using the game, some variables were collected and stored by the game itself.

The application setup was composed of an individually prepared room for each participant, with Kinect 360 for Windows^®^ connected to a Windows 7 laptop, and the A&R exergame pre-installed. The game was displayed through a projector on a big screen. A research assistant was available to help the participants in every session.

### Participants

The inclusion criteria for participants were: absence of genetic, psychiatric or neurological disorders; absence of uncorrected sensory disabilities; hadn’t scored below the 25th percentile in the Raven Colored Progressive Matrices test (Portuguese translated version – Angelini et al., 1999).

Only students who attended more than 70% of the game sessions had their data collected and taken for analysis. According to this rule, no child was excluded from the sample. Ethical aspects were also considered in the project, which was submitted and approved by the university’s ethics committee. The children’s parents also authorized their participation in the research. The participants were composed of five boys and two girls with a mean age of 7.86 (1.46) years old. The average socioeconomic status of the participants was classified as B1 (ABEP, 2014). Three children were in their first year of primary school, whereas two were in their third year of primary school. The other two were in the fourth year of primary school.

### Instruments

The following data from the participants were stored during the pilot study: Name, sex, age, and school year. Moreover, data related to the game use were collected, namely: activity, timestamp, level type, and performance. All variables were considered in the analyses.

To evaluate the association of quantitative variables with performance, Spearman’s correlation (Hollander and Wolfe, 1999) was used, and Mixed Effects Logistic Regression (Fitzmaurice et al., 2011) was employed to identify aspects that influenced performance, with the subsequent use of the Backward method (Efroymson, 1960) for the selection of the significant variables. The analyses based on the data were the following:

1. Exploratory analysis of students’ performance, which aims at evaluating the performance of the participants in the activities, as well as the average performance in each minigame and each level type.
2. Exploratory analysis of success, which aims at identifying the chances of participants to succeed in each level type based on descriptive analysis.
3. Analysis of the influencing factors on student’s performances during the activities, using Mixed Effects Logistic Regression to verify the dependent variables and random effects, applying the Backward method to select the significant variables.

## Analyses and Results

Within the exploratory analyses of the variables of interest, the performance of the students is considered to have a number between 0 and 1, with 0 being the lowest value and 1 the highest. Therefore, a performance of 0.90 indicates the overcoming of 90% of the challenges in a certain level of the game.

### Exploratory Performance and Success Probability Analysis

**FIGURE 2:**
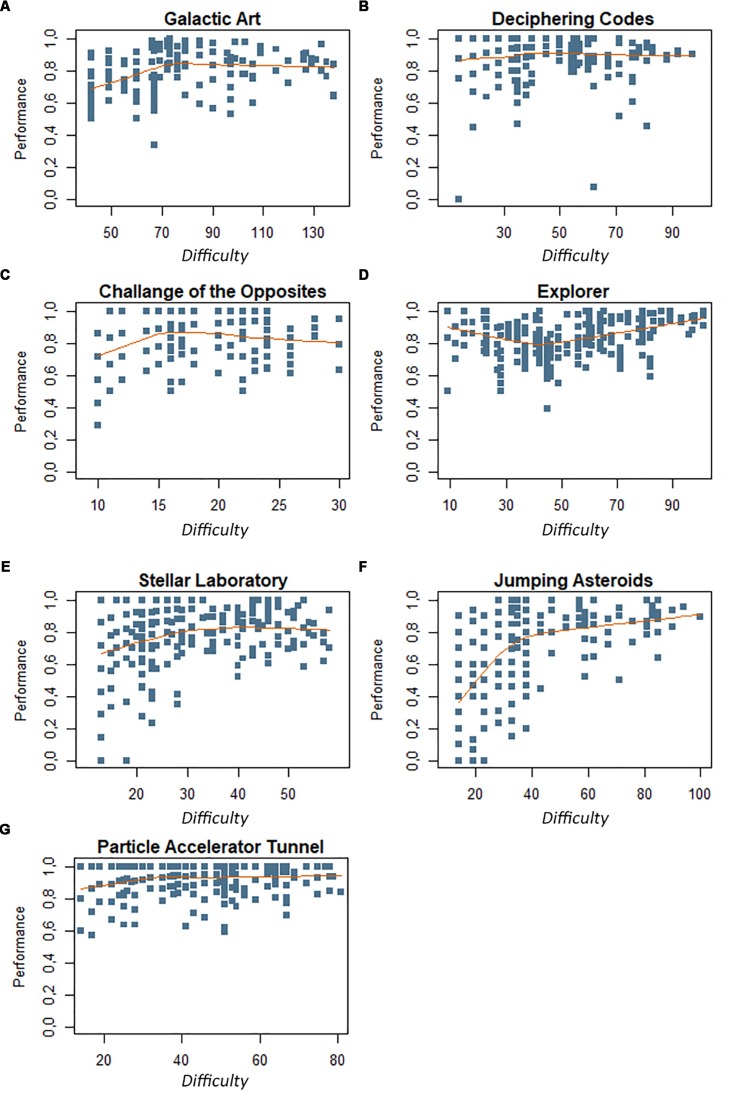
**(A–G)** Performance concerning the difficulty of the activities. The participant’s performance registered by the game is depicted in the orange range. Each activity level is represented by its inherent difficulty in the lower row, e.g., Level 1 of the Particle Accelerator Tunnel activity has a total of 14.2 difficulties, hence all the blue squares in that column represent the performance of the participants in that level. The orange line represents the average participant’s performance. It is noteworthy that more than one participant performance is registered in a single blue square.

For the exploratory performance analysis of the seven participants in the 1528 rounds played, Spearman’s correlation between performance and difficulty levels for each activity was: **Galactic Art** (Figure 2A) (*n* = 144) (*r* = 0.25, *p*-value = 0.002); **Challenge of the Cosmic Opposites** (Figure 2C) (*n* = 171) (*r* = 0.15, *p*-value = 0.051); **Explorer** (Figure 2D) (*n* = 283) (*r* = 0.17, *p*-value = 0.003); **Stellar Laboratory** (Figure 2E) (*n* = 280) (*r* = 0.33, *p*-value = 0.000); **Jumping Asteroids** (Figure 2F) (*n* = 214) (*r* = 0.56, *p*-value = 0.000); **Particle Accelerator Tunnel** (Figure 2G) (*n* = 250) (*r* = 0.19, *p*-value = 0.003); and **Deciphering Codes** (Figure 2B) (*n* = 186) (*r* = 0.11, *p*-value = 0.148).

In general, the correlation between performance and difficulty was significant and positive, which means that the greater the difficulty, the greater the performance of the player. It is important to emphasize that the first stages, from levels 1 to 9 in Figure 2, presented some below-average performance values, which may have been produced because the children were learning to play a new game.

**FIGURE 3:**
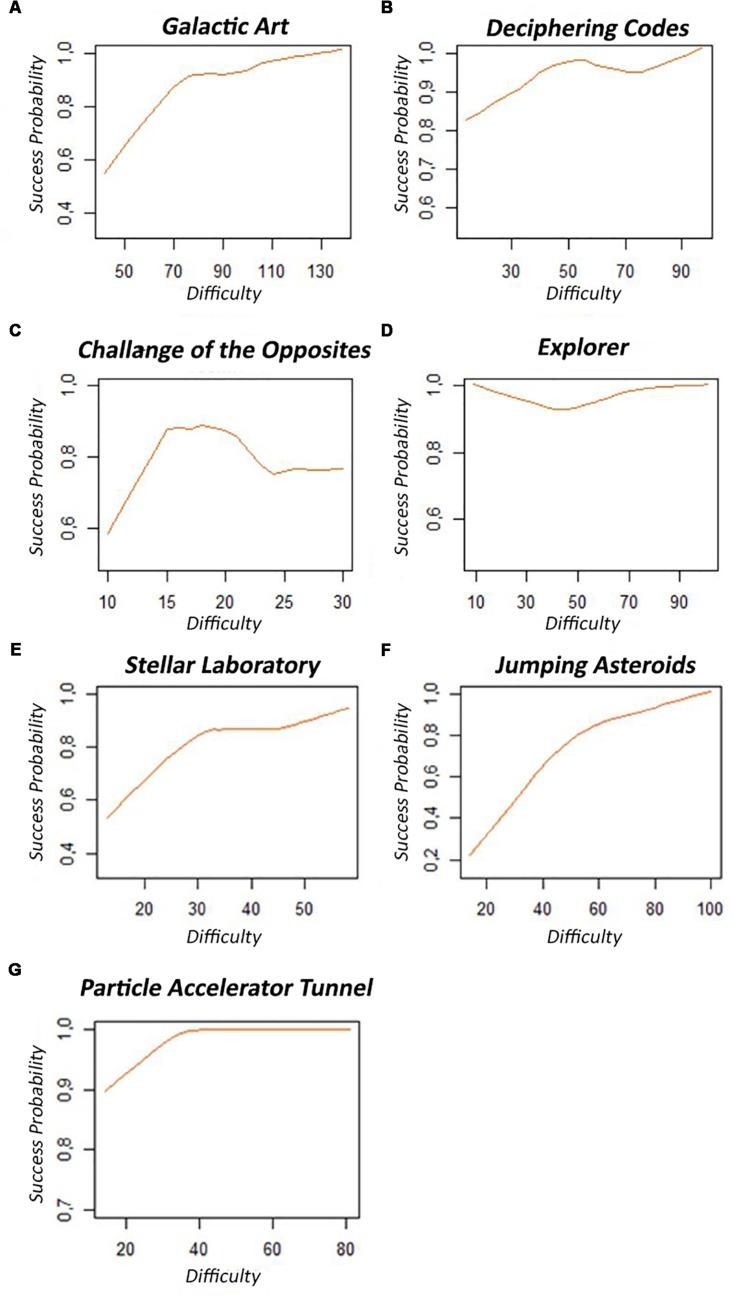
**(A–G)** Difficulty related to the probability of success for each minigame. Each activity level is represented by its inherent difficulty in the lower row, and the success probability in that difficulty is depicted as the orange line until the last player entry registered in the game.

An exploratory analysis of the variables of interest concerning the students’ probability of success in the activities was also carried out. In this context, whenever the player reached the performance of at least 70% in a certain level, he would win. Hence, each level had an associated difficulty, for which success was a binary information (value = 1[successful]; value = 0[unsuccessful]). Figure 3 shows that in most activities it was possible to observe that the initial levels had a low success rate and, as the player advanced in the stages, the success rate increased, even if the difficulty level also increased. Thus, one can infer that the player was progressively learning to overcome the challenges presented in the minigame.

### Analysis of the Factors That Influenced the Player’s Performance

Mixed Effects Logistic Regression (Fitzmaurice et al., 2011) was used to identify variables that influenced performance of the seven participants in the 1528 rounds played, considering student data (age, sex, school year), game level, and the difficulty associated with each level of the minigames. Subsequently, the Backward method was applied (Efroymson, 1960), which identified as significantly influential in the performance the following: school year and game level, as shown in Table 1. A 5% significance level was adopted for the Backward method. R software was used.

**TABLE 1:** Mixed effects logistic regression for performance.

**Variables**	**Final model**		
	***p*-value**	**OR**	**CI – 95%**
Age	–	–	–
Sex: Girls	–	–	–
Sex: Boys	–	–	–
School grade: first grade	–	1	–
School grade: third grade	0.078	1.46	[0.96;2.21]
School grade: fourth grade	0.000	2.39	[1.57;3.63]
Type: Rest	–	1	–
Type: Normal	0.000	0.71	[0.59;0.86]
Type: Peak	0.001	0.62	[0.47;0.83]

*The OR columns stand for Chance Ratio, that is, the ratio between the possibility of an event to occur in one group and the possibility of the same event to occur in another group. The CI – 95% column (95% Confidence Interval) ensures that the estimated parameter is within this range in other samples from the same population.*

Table 1 shows that there was a significant difference (*p*-value = 0.001) between the **Peak** level type compared to the **Rest** level type, considering students with similar capacity who played the same game at the same level. The performances in the **Peak** level type were lower when compared to the **Rest** level type since the students who were in the **Peak** level type had a chance 0.62 times lower to match the challenge predicted in the level type than the students who were in the **Rest** level type (0.47;0.83). The **Normal** level type differed significantly (*p*-value = 0.000) from the **Rest** type. Students who played the **Normal** level type had a 0.71 times lower chance to succeed in predicted challenges than the students who played the **Rest** level type (0.59;0.86). Therefore, the performance in the **Normal** level type was lower when compared to the **Rest** level type.

These findings confirm the planned behavior of the DCs depicted in Figure 1. The controlled difference of the difficulties allowed the presentation and regulation of the type and number of challenges that the student had to face in each level of the activities. Thus, the alternation between the level types (Normal/Peak/Rest) helped the players to avoid the comfort zone. This can be observed in Table 1 Chance Ratio of accomplishing tasks in the Rest type levels, which were higher when compared to the Normal and Peak level types.

## Discussion

As presented in section “Development and Quantification of the Difficulty Curves,” different criteria had been established to model the DC and make the game more attractive, engaging, and fun, proposing challenges that matched the players’ skills. It is important to highlight the relevance of computing the DCs during the game development process as the difficulties must be assessed and their weights assigned according to the GM (Schell, 2008; McMillan, 2013). Thus, considering that the GM and their complexities change from game to game, the definition and assignment of values for the curves must be tested by game designers, specialists and more importantly, the target audience, to validate the GM’s weights. The evaluation stage mentioned in section “Development and Quantification of the Difficulty Curves” (Mossmann et al., 2017) also contributed to present the player with new and more complex challenges considering his/her previous learning, thus, producing a cyclic (periodic) balance between challenge and skills (Cowley et al., 2008). As indicated by the pilot study results, most of the DCs showed a significant and positive Spearman correlation between the difficulty levels and the participants’ performance. Therefore, the minigames in A&R may produce an environment that favors the player to reach the Flow state, which is desirable in games for IC stimulation designed for children.

The minigames were composed of different challenges and difficulties lined by rules so that it presented the player with challenges that they were able to overcome. This feature for conducting the player to the Flow state was described by Cowley et al. (2008) and was implemented here according to the details presented in section “Model Application.”

Furthermore, there must be a balance between the challenges presented in the game and the person’s ability to overcome these challenges (Schell, 2008). The players are expected to practice and exercise the tasks in the game throughout the game levels, thereby perfecting their skills and learning to overcome challenges (McMillan, 2013). Thus, while going through each game level, the students improved their abilities. As the level difficulties increased, the players’ performances also improved, according to the general positive Spearman’s correlation computed.

Cowley et al. (2008) indicated that the DC of a game must establish a link between the player’s (intrinsic) ability and the external challenges inherent to the game (extrinsic to the player). Besides, the player must be (intrinsically) interested, willing and able to learn and improve his skills. This must match the game system, which must be designed to identify the player’s skills, presenting challenges that are consonant to each player (Schell, 2008). To offer the children appropriate challenges, the minigames developed in this research were planned to have increasing difficulty levels and no final stage. Thus, if the player demonstrated abilities greater than the challenge, he could quickly go through the easier levels and find the appropriate challenges at advanced levels. According to the data in Table 1, students in higher school years were able to overcome the initial challenges more quickly and could face challenges consistent with their skills at advanced levels, since the DCs presented increasingly difficult challenges.

Nevertheless, further experimental investigations are needed to estimate if the DCs planned in this study can be adapted considering the results obtained according to the players’ school year (Table 1).

Furthermore, the graphs in Figure 3 illustrated the success probability of a minigame. They show that, in general, the greater the difficulty, the greater the expectation of success. Therefore, the students improved their abilities by playing through subsequent game levels, which increased their chances of success. The same result was found in Spearman’s correlation of performance data, shown in Figure 2. Accordingly, the challenges were relevant to the improvement of the player’s ability.

The DCs planned for the minigames worked as expected in general, enabling the selection of more complex challenges considering the player’s previous learning phase. This made it possible to keep a balance between the challenges and the children’s abilities shown in varying level types (Normal/Peak/Rest) as explained in Figure 1. The model presented in Table 1 shows that the players had a worse performance in the Peak level when compared to the Rest level, as the chance ratio to succeed the challenges in the Peak levels was smaller than in the Rest stages. Furthermore, the Normal levels also presented lower chance ratios to succeed in the challenges when compared to the Rest levels.

Ultimately, the DCs of the game achieved satisfactory results in terms of players’ performance and success based on the previous game evaluation. That demonstrates the children’s understanding of the activities and their evolution in the cognitive stimulation activities, previously approved by neuropsychology experts. The performance of the DCs in the activities allowed the players to engage in the game, which shows the potential of the proposed approach for the development of digital games tailored to IC stimulation in the future.

To enhance their cognitive stimulation, players must perform tasks that demand and train their executive skills according to their abilities (Cowley et al., 2008). The DCs in a stimulation game require the tasks to be planned in a way that enables the players to continue to have a game experience that does not tend to indifference or anguish, according to the Flow model. Therefore, the main contribution of this work is in the field of game development for cognitive stimulation.

The most important aspects of this research are the following: (a) It contributes to the development of games directed to IC stimulation, with emphasis on the use of the Flow model as a paradigm to influence people’s participation in given activities; (b) The study points to an intersection among fields such as neuropsychology, computer science, education, and digital games.

Nevertheless, we are aware that our research may have three limitations: (a) The scope of this article does not address the impacts of pre/post neuropsychological tests performed by the participants, results that will be published in future papers; (b) DCs weren’t meant to fit each player, causing a more skilfull player to take longer to reach a challenging level. Concerning the school years, a curve should be considered for each school year in future works to optimize a possible gain in IC; (c) This was a pilot study that involved only seven participants of three different school grades. Future research should focus on different school grades separately, also involving a higher number of participants.

This research was conducted with the aim to contribute to discussions in the field of IC stimulation with digital games, by approaching game design techniques as one of the parameters for the development of stimulation activities. In doing so, the use of the model for the definition of the A&R DCs produced overall satisfactory results in the performance and probability of success with the target audience. Besides, our findings highlight the relevance of games’ DCs as cognitive enhancement outcomes in neuropsychological and educational interventions, in addition to standardized neuropsychological tools. Finally, we expect that the development of cognitive stimulation digital games, through the Flow-oriented difficulty computation parameter, makes them more fun, interesting and engaging for their users.

## Data Availability Statement

The datasets generated for this study are available on request to the corresponding author.

## Ethics Statement

This study was carried out in accordance with the recommendations of Resolution CNS 466/12, under the CAAE no. 58350416.0.0000.5336, Research Ethics Committee of the Pontifical Catholic University of Rio Grande do Sul with written informed consent from all subjects and their parents. All subjects gave written informed consent in accordance with the Declaration of Helsinki. The protocol was approved by the Research Ethics Committee of the Pontifical Catholic University of Rio Grande do Sul.

## Author Contributions

JM, ER, and RF designed the study. JM and RF prepared the experimental materials. JM, RF, ER, and BC carried out the data collection and the statistical analyses. BC wrote the first and second draft of the manuscript. DB, JM, RF, and ER provided revisions and approved the final version.

## Conflict of Interest

The authors declare that the research was conducted in the absence of any commercial or financial relationships that could be construed as a potential conflict of interest.

## Footnotes

1 Game Mechanics (GM): possibilities, behaviors, and elements for the player to interact and overcome challenges in a determined game (Hunicke et al., 2004), e.g., in a car racing game, the player can accelerate, brake, and orientate the car. Each of these are the fundamental GM for the player to drive. However, other GM could add difficulty, such as rainy or desert races.
