# Supplementary material for: The Planning of Difficulty Curves in an Exergame for Inhibitory Control Stimulation in a School Intervention Program: A Pilot Study
Source: Front Psychol. 2019 Oct 15;10:2271. doi: 10.3389/fpsyg.2019.02271 (PMC6804572; doi:10.3389/fpsyg.2019.02271)
Supplement: Supplementary file 1 [file Table_1.docx]

Supplementary Material

# Supplementary Figures and Tables

## Supplementary Tables

|  | Speed Weight (w1) = 4 | Obstacle Quantity  Weight  (w2) = 1 | Inverted Camera Distance  Weight (w3) = 2 | Reverse Obstacle Quantity  Weight (w4) = 3 |  |
| --- | --- | --- | --- | --- | --- |
|  |  |  |  |  |  |
| Level | Value (v1) | Value (v2) | Value (v3) | Value (v4) | Total Difficulty (TD) |
| 1 | 1.1 | 5 | 0.9 | 1 | 14.2 |
| 2 | 1.2 | 7 | 0.9 | 1 | 16.6 |
| 3 | 1.3 | 9 | 1 | 1 | 19.2 |
| 4 | 1.3 | 9 | 1 | 2 | 22.2 |
| 5 | 1.4 | 11 | 1.1 | 2 | 24.8 |
| 6 | 1.4 | 11 | 1.1 | 3 | 27.8 |
| 7 | 1.2 | 13 | 1.2 | 2 | 26.2 |
| 8 | 1.2 | 12 | 1 | 2 | 24.8 |
| 9 | 1.2 | 11 | 1 | 2 | 23.8 |

**Supplementary Table 1.** Difficulty Curve variables computed for the Particle Accelerator Tunnel activity.
